# Towards Equitable Health Care Access: Community Participatory Research Exploring Unmet Health Care Needs of Homeless Individuals

**DOI:** 10.1177/08445621211032136

**Published:** 2021-08-13

**Authors:** Melba Sheila D’Souza, Noeman Ahmad Mirza

**Affiliations:** 1 School of Nursing, 33527 Thompson Rivers University, Kamloops, BC, Canada; 2 Faculty of Nursing, 8637 University of Windsor, Toldo Health Education Centre, Windsor, ON, Canada

**Keywords:** homeless, access, health services, primary care, community, qualitative research

## Abstract

Community and health services often overlook health care needs of persons experiencing homelessness, which leads to deterioration in health and increased utilization of emergency services. Since homeless people are underrepresented in health service research, little is known about their unmet health care needs, particularly in smaller cities where resources are limited. This community-based participatory research explored the experiences of small-city homeless service users (HSUs) with unmet health care needs and community service providers (CSPs) who work with them to determine barriers to health care access affecting them. Structured interviews were conducted with 65 HSUs and 15 CSPs in interior British Columbia, Canada. These interviews were audio-recorded, and the retrieved data were analyzed thematically. The three themes uncovered included: a lack of access to health care and risk of stigma, a lack of trust and fear of discrimination, and a need for community navigation and social support. The findings indicate that HSUs do not receive equitable care and face challenges in accessing appropriate and timely foot care, which contributes to increased foot-related emergency visits, loss of trust in the health care system, and disabilities due to worsening foot conditions. Various social determinants of health also impact HSUs, such as low socioeconomic status, lack of housing, decreased social support, unhealthy behaviors, and inequitable access to health services. By collaborating with HSUs, community and health services need to develop innovative outreach programs that provide better community resources as the first step toward equitable access to health care.

## Introduction

Homeless individuals live with complex health conditions and multimorbidity including worsening health conditions; and have a difficult time receiving community support and accessing health care for their unmet health care needs. Foot care services provide early screening, detection, prevention, and treatment of foot problems. In British Columbia (BC), Canada, general medical services often end up providing specialized foot care to homeless individuals. However, these services are more prominent in large city centers. In small cities (population of 100,000) that also serve suburban and rural areas, foot care services are underresourced.

There is very little known about the unmet health care needs of homeless individuals residing in small cities with limited specialized health services. Anecdotal evidence shows that homeless individuals often access general care through outreach and mobile service centers, but their foot care needs are often left unattended. In the absence of research evidence on the unmet foot care needs of homeless individuals, health care systems face challenges in determining the appropriate services to be developed to address the health care needs of homeless service users (HSUs). To address this gap in the research, this study reports on a qualitative study on the experiences of HSU with unmet foot care needs and the community service providers (CSPs) who work with them in a small city in interior BC, Canada.

## Background

Homelessness is a social issue in which an individual or family lives without stable, permanent, appropriate housing or the immediate prospect of such being acquired (Barnaby et al., 2010). It can result from a lack of affordable, suitable housing, and the individual or household's financial, mental, cognitive, behavioral, or physical challenges (Gaetz et al., 2014; Gaetz et al., 2018). Homeless individuals living in motels, community placements, and institutionalized care may have supportive care from social and community services. However, when they leave these places without any plans for an adequate safe dwelling, they are left to meet their basic needs on their own and are at risk of becoming homeless again (Kidd et al., 2017; Martin-Fernandez et al., 2018).

Homeless people often need to survive on the streets, with low income, lack of shelter, food insecurity, poor mental health, and potential violent attacks. This puts them at a higher risk for mortality, morbidity, and hospitalization compared with those who have some form of shelter and a source of income assistance (Maness & Khan, 2014). Homeless individuals usually experience several physical health problems as well, such as hypertension, diabetes, respiratory and musculoskeletal disorders, infectious diseases, cognitive and behavioral challenges, and worsening skin and foot problems (Gerber, 2013; Woolley, 2015).

Lack of health insurance, cognitive impairment, abuse, alcohol- and drug-related problems, traumatic brain injury also affects this population (Argintaru et al., 2013; Nikoo et al., 2014; Saddichha et al., 2014). This impacts access to health care services for a person who is homeless. While health care services can address certain health care concerns of individuals experiencing homelessness, there is, however, a lack of services that cater to their foot-related health care needs (Maness & Khan, 2014).

It is estimated that 35,000 Canadians experience homelessness on any given night, and at least 235,000 Canadians are homeless in any given year (Rech, 2019). In 2018, 25,216 people were reported to have experienced absolute homelessness (unsheltered and living on streets), with another 6,789 in transitional (temporary) homes in Canada (Employment and Social Development Canada, 2018). According to the Employment and Social Development Canada (2018), people experiencing chronic homelessness (on the streets for months and years) account for 60% of all respondents who are homeless, whereas episodic homelessness (moving in and out of homelessness for short intervals) account for 8% of all respondents who are homeless across 61 communities across Canada.

Statistics on homeless individuals further show that 28%–34% of the shelter population is indigenous, 27.3% are women, and 18.7% are youth (Gaetz et al., 2016). An examination of the social determinants of health shows that in BC, high rents, low incomes, and unavailable housing are indicated to be the common causes of homelessness (The Homelessness Services Association of BC et al., 2018). As a result, homeless individuals must couch surf (moving from one temporary housing arrangement to another) or rely on live-in emergency shelters, transitional homes, alleys, or vehicles.

When homeless individuals experience health problems, they are usually unable to access social and health care services due to lack of health insurance, competing needs, housing instability, decreased mobility, long wait times, and discrimination (Hoshide et al., 2011; Hwang et al., 2011; Winetrobe et al., 2016). Structural factors like poverty, discrimination, lack of affordable housing, and impact of colonialism on indigenous peoples continue to affect people's experience of homelessness (Gaetz & Dej, 2017). Physical health conditions along with comorbidities such as mental health and substance use present barriers to adequate care (Foster et al., 2010). High rates of mental health disorders like alcohol and drug dependence, depressive disorders, posttraumatic stress disorder, head injury, hepatitis B or C, and psychological traumatic experiences are prominent among homeless individuals (Krausz & Schuetz, 2013).

Homeless individuals living in emergency shelters and on the streets experience a range of skin, foot care, and chronic pain management challenges that impact their health, well-being, and ability to walk and perform activities of daily living (Lamanna et al., 2018; Zlotnick et al., 2013). Common foot concerns reported by homeless individuals include bunions, hammertoes, gout, plantar warts, foot ulcers, and frostbite (To et al., 2016), as well as diabetic foot ulcers, corn and calluses, nail pathologies, foot infections, and injuries (Chen et al., 2014; Chong et al., 2014; Matteoli et al., 2015; To et al., 2016). However, foot screening services are mainly located in large city centers, and there continues to be a need for such screening services in small cities so adequate health promotion strategies could be developed for homeless individuals with unmet foot care needs.

## Conceptual framework

**Figure 1.**
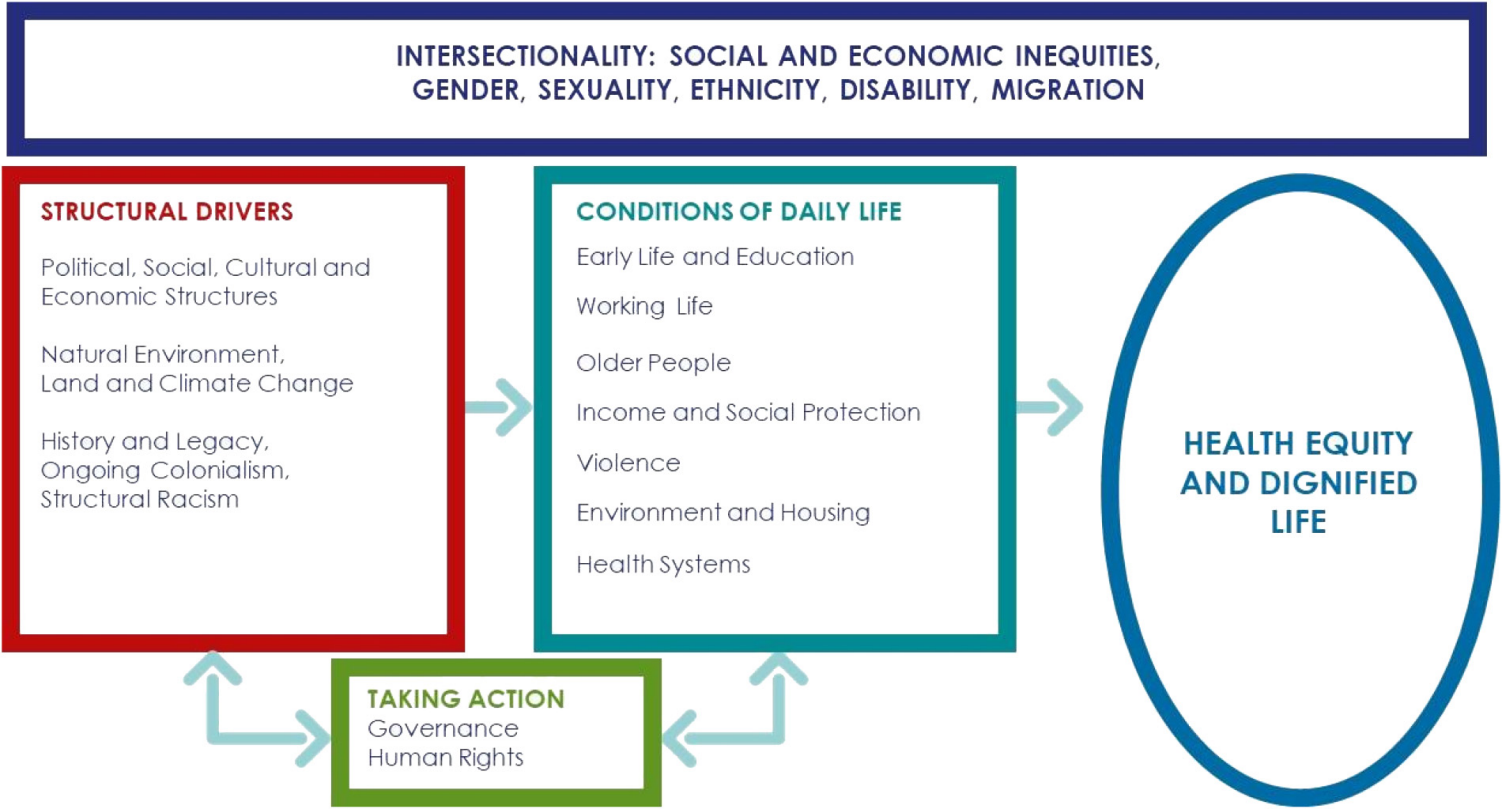
Conceptual framework on social determinants of health and health equity (Pan American Health Organization, 2019).

Systems failures that contribute to homelessness occur with barriers to accessing publicly funded services, inadequate transitions from publicly funded institutions, and gaps within and between community and health systems (Gaetz & Dej, 2017). To examine unmet foot care needs of homeless individuals, we used a Social Determinants of Health and Health Equity framework (SDHHE; Pan American Health Organization, 2019). The conceptual framework (Figure 1) on a social determinant of health framework with a health equity emphasizes the concepts and interactions between structural racism, importance of relationships to people, and inequities in health care (Pan American Health Organization, 2019). It assumes that the conditions of daily life and the systems in place are shaped by the socioeconomic and cultural context which highlights the role that these structural factors have on the health of service users. The quality of these conditions affects people's social connection and influences their health. Hence, there is a need to provide adequate support to build capacity to help them meet their health care needs (Pan American Health Organization, 2019).

## Literature

Many foot problems experienced by HSUs are caused by the lack of proper footwear, repetitive trauma, prolonged walking and standing, and extensive exposure to moisture and cold weather (Hwang et al., 2011; Lipato, 2012; Muirhead et al., 2011). In a study involving 299 homeless individuals, many reported standings for long hours on their feet. More than half experienced foot pain, and one in five had edema, neuropathic symptoms, and reported dermatologic foot problems (Chen et al., 2014). These problems are known to lead to multimorbidity, increased hospitalization, and disability (Matricciani & Jones, 2015). The extent and severity of foot problems usually depend on an individual's use of alcohol, nicotine, and drugs; poor nutrition; dehydration; diabetes; hypothyroidism; peripheral vascular disease; and peripheral neuropathy (Thomas, 2019).

While HSUs are usually seen in general medical clinics and emergency departments for their foot concerns, most of them require follow-up due to foot problems (Chen et al., 2014). However, many choose not to access health care services even when they require urgent medical attention (Thomas, 2019). Many HSUs often feel neglected because of health care professionals’ attitudes based on the stereotypes that portray homeless individuals as people who are addicted to drugs and have mental health conditions (Campbell et al., 2015). For some homeless individuals, their foot attire (e.g., shoes and socks) and foot odors prevent them from seeking foot care services (Muirhead et al., 2011). For others, it is the stigma and discrimination they face in health care facilities that negatively affect their decision to seek medical help (Dej et al., 2020; Johnstone et al., 2015; Mejia-Lanchros et al., 2020).

In a study involving 497 people who were homeless from the three health authorities of BC (Island, Coastal, and Northern), 63.2% reported having access to a regular medical doctor or nurse practitioner. In addition, 34% felt that there was a time over the past year they needed care but could not receive it (Krausz et al., 2013). These numbers were lower than the nationwide average of 85.5% and the 82.3% in BC who reported having access to a regular health care provider in 2019 (Statistics Canada, 2019). While Krausz et al.’s (2013) study focused on three BC health authorities, no data were collected from the regional health authority Interior Health. In interior BC, two small cities (population: 90,000 to 132,000) act as health care hubs. Health care options and resources are limited for an individual who is experiencing homelessness. As a result, this individual must often resort to emergency services for unmet health care needs that could otherwise be better addressed through primary and community care.

In Chen et al.’s (2012) literature review on several qualitative studies focusing on the general health of homeless individuals, only three studies addressed their podiatric or foot care needs. Another qualitative study by Martin-Fernandez et al. (2018), which involved 15 homeless individuals, showed that homeless individuals experienced compromised health due to the lack of food, shelter, and clothing. This study did not focus on foot care needs. Similarly, Alecrim et al.’s (2016) interview study with six service providers also focused on general health service provision. Homeless individuals’ hesitation for follow-up care signals the need for more institutional and social resources and incentives to keep sick HSUs in treatment.

The existing literature on the health and well-being of HSUs has focused mainly on their general health and mental health care needs; but has overlooked their unmet foot care needs and the social barriers they experience in accessing health care services for their foot conditions. Despite the lack of qualitative studies that focus on homeless individuals’ unmet foot care needs, Kapetanovic (2016) conducted qualitative research on the barriers and facilitators that 26 HSUs experienced when accessing general health care services in the United States. These barriers included limited income, lack of healthy food availability, lack of access to primary care physicians, and waiting on entitlement decisions. Meanwhile, the facilitators included local community resources, access to health care services, counseling, shelters, military benefits, and state entitlement programs. While this study examined barriers and facilitators related to accessing general health care services, there remains a need for qualitative research on the barriers experienced by HSUs with unmet needs.

## Aim

To understand the barriers HSUs experience when accessing health care services for their foot conditions, we conducted a qualitative study that explored the experiences of HSUs with unmet foot care needs and the CSPs who work with them in a small city in interior BC, Canada.

## Methods and Procedures

### Ethics Considerations

This study was reviewed and approved by the relevant institutional research ethics board. The participants were free to withdraw their participation at any time, without consequences to the services they were receiving. All participants received an information letter describing the study and participant expectations, the need for informed consent, and that they would be given the opportunity to ask questions at any time. Confidentiality and participants’ privacy were maintained and safeguarded throughout the study. Data anonymity was assured by assigning code names, such as AA001, AB002, AC003, and so on, during the data entry, coding, and transcription processes. This aided in anonymizing the data so that study participants could not be identified.

### Setting and Design

Community-oriented participatory research (COPR) involves partnerships between academic and community organizations with the goal of increasing the value of the research product and acknowledging the participant's needs (Coughlin & Smith. 2017). The COPR approach highlights community resilience, resources, and opportunities for growth rather than focusing on health problems. COPR approach explores the barriers HSUs experience when accessing health care services in one of two small city hubs in interior BC, Canada. The shared collaboration included community and academic partners to engage HSUs and explore contextual factors and foot care challenges. Community stakeholders offered various forms of community support, such as housing, shelters, medical aid, social work, an outreach center, living accommodation, rehabilitation, a kitchen, and a health center initiative for HSUs. This collaborative team developed partnerships, validated findings, and engaged in knowledge mobilization.

### Sampling

This study used a purposive sample comprising of 65 HSUs from different shelters, and housing and drop-in centers; as well as 15 CSPs who worked directly with HSUs in the community. Eligible HSUs were defined as adults who were above 18 years of age, sheltered in transitional houses, spoke English, were able to provide written informed consent, and could participate voluntarily. Vulnerable persons who were experiencing domestic violence, abuse, active mental health problems, or cognitive impairment were excluded from the study. Eligible CSPs were defined as adults who were above 18 years, worked directly with HSUs in transitional houses, spoke English, were able to provide written informed consent, and participated voluntarily.

### Development of Data Collection Tools

By consulting the literature, the CSPs, and HSUs, we developed a demographic questionnaire to collect baseline information about HSUs. The demographic form included questions on respondents’ demographic and socioeconomic background. We also developed a structured interview guide for HSUs. This interview guide (Table 1) included questions on foot experiences, walking concerns and duration, coping, foot problems, footwear, and foot hygiene. Similarly, we developed a structured interview guide for CSPs which included questions on foot care services, foot concerns, foot practices, foot care strategies, health care services, and social services. We had two CSPs, two HSUs, and one registered nurse independently review each of the tool's contents to determine their validity for comprehension and appropriateness. The modified data collection tools were pre-tested with two HSUs and three CSPs to assess dependability.

**Table 1.** Interview Question Guide for Study Participants.

Homeless service users
1. How do you feel and how have things been with you during the past 4 weeks?
2. How do you feel you are coping with your health concerns or care?
3. Tell me about a health care experience that stands out in our mind.
4. Tell me about the time when you got care at a hospital, emergency room, or clinic.
5. What challenges have you experienced with your health care needs?
6. What health concerns or experiences do you have in the past or the present?
7. What needs or problems do you have with your health care services?
Community service providers
1) What changes would be helpful to make access to health care easier for the homeless?
2) What do you feel is important about your experiences serving a person who is homeless?
3) What kind of health care services are used by adults who are homeless?
4) What kind of health concerns do you see with individuals who are homeless?
5) What kind of social support is provided for a person who is homeless?
6) What kind of strategies are needed to meet health care of the population who are homeless?
7) What types of programs and services are essential to support a person who is homeless?

### Data Collection

Interviewers spent 40–50 min on each interview and used a structured interview protocol that included an introduction, informed consent process, demographic and open-ended questions, and a debriefing session. Interviewers also prepared field notes of the protocol and used a reflective journal. The principal investigator spent 2 days a week for 6 months in community organizations for the data collection. Privacy, dignity, and comfort were ensured with a private space during the interviews. Data collection took place between September 2019 and February 2020.

### Data Analysis

All 80 interviews with 65 HSUs and 15 CSPs were cleaned, deidentified, and transcribed verbatim by the primary investigator onto NVivo 9, a qualitative data analysis software. We identified key concepts using content analysis, reviewed, and coded the transcripts. We compared the perspectives of the participants by role.

To analyze the data, we used Braun and Clarke (2006) qualitative analysis method. First, we read and reread the interview transcripts to familiarize ourselves with the data. Second, we established a coding template by using consistencies and divergences between diverse participant perspectives. This transcription helped us to generate codes and outline patterns and categories to compare within and across all transcripts. Third, we iteratively identified the themes from the participants’ quotations. This process involved interpreting the findings, looking for patterns, and finalizing the themes. Fourth, we validated the study findings with two HSUs, three CSPs, and three community stakeholders. This final step acted as a type of member-checking stage to increase the data's trustworthiness.

## Results

### Demographic Data

Men comprised 69.2% of the participants, women 30.8%. Of the participants, 60% had completed middle school and 20% were in high school (Table 2). About 38.5% reported a history of health problems and emergency visits. Participants reported that they had access to adequate housing (55.38%), emergency services (46.15%) urgent health care (32.31%), and foot care clinics (7.69%). Sixty percent were above 50 years of age, and 60% experienced chronic (more than 5 years) homelessness.

**Table 2.** Demographic Characteristics and Access Among Service Users *N* = 65.

Demographic	Variable categories	Frequency (percentage)
Age	More than 50 year	26 (40%)
	Less than 50 years	39 (60%)
Age (mean + *SD*)		52.08 + 9.86
Years of homelessness	More than 5 years	26 (40%)
	Less than 5 years	39 (60%)
Gender	Male	45 (69.2%)
	Female	20 (30.8%)
Years of schooling	Grade 8: Middle school	39 (60%)
	Up to Grade 11: High school	13 (20%)
	Grade 12: Higher secondary, Vocational/Diploma	13 (20%)
History of health problems	Yes, Health problems	25 (38.5%)
	Fewer health problems	40 (61.5%)
Frequency of emergency visits	More than 5 times a year	25 (38.5%)
	Less than 5 times a year	40 (61.5%)
Access and utilization of community services	Food programs, soup kitchens	42 (64.62%)
	Adequate, safe housing	36 (55.38%)
	Steady employment, income	10 (15.38%)
	Mental health, addiction clinics	34 (52.31%)
	Emergency, urgent health care	30 (46.15%)
	Primary care providers, walk-in clinics	21 (32.31%)
	Foot care, wound care clinics	5 (7.69%)

### Themes

We identified and validated three themes based on the collected data: (1) lack of access to health care and risk of stigma, (2) lack of trust and fear of discrimination, and (3) need for community navigation and social support.

#### Theme 1: Lack of Access to Health Care and Risk of Stigma

During the focused interviews, CSPs stated that HSUs access the following health care services in order of mostly accessed to least accessed: the emergency room, walk-in clinics, street nurses, wound care clinic, community clinics, family doctors, mobile injection sites, and nurse practitioners. However, HSUs have minimal access to these services. One HSU stated that “*I do not have family doctors or nurse practitioners. They just wait in line in the emergency room.*” [HSU38]. While there is a wound care clinic, one HSU said:

I waited for hours before finally the nurse or doctor saw me. If they [homeless SUs] do not have a referral, they cannot be seen at the wound care clinic, despite having a bad infected wound. So, they must go to the emergency. If they have a friend who can take them to the emergency, it works out; otherwise, they must wait it out. It is hard to get over it. I do not go to the emergency because of my previous experience, I do not trust them. [HSU41]

HSUs mentioned that they visited the emergency room or nearby urgent care clinics because they did not have a primary care physician. When some participants visited the emergency department, they had a negative care experience attributed to their self-identity and the stigma. One HSU recalled:

There was this one time I visited the emergency for my foot problem and old back injury. I wanted to get it [my foot] checked, but the doctor checked my blood pressure and asked me a few questions and signed my papers. I did not get any pain medications or anything from this [for the foot problem]. He [the doctor] just did not look at me [avoided eye contact throughout the examination] because I am different. I have seen stigma and racism. I felt uncomfortable. Sometimes I cannot get the medicines, they are expensive. [HSU19]

Another HSU who had previously had an addiction problem stated:

Someone ran a motorbike over my right foot. It still hurts me, and I think the bone may be broken. I ended up in the emergency room. I did not get much help, at least the help I hoped for. The main thing I wanted was painkillers to make the foot pain go away. I did not even get Percocet or Dilaudid, you know? He [the physician] did not want to give me any pain medications [for my foot problem], just gave me Tylenol and a Voltaren. It seems like they already know that I am homeless, with a token. They are judgemental, there's discrimination. These medications do not help [my foot problem], I have had a pinched nerve in my foot which gives me constant pain and makes my foot fall asleep [lack of sensation, numb, pins and needles]. [HSU4]

Some HSU shared how challenging it was for them to access health care services and cope with their chronic health problems. One HSU stated:

I have diabetes and high blood pressure, but getting my medication is hard. I am trying to get better on the streets. I go to the ER when I need my medications. They [staff] ask me to have you had any alcohol or are you taking drugs. I do not have enough money to pay for some of them [e.g., insulin pump, pen insulin, insulin cartridges, blood glucose monitors, strips, lancets. I did not have a vehicle [transportation] to get to the emergency room. [HSU9]

With the access and availability of health care, some SUs felt they could not get the health care they needed, mostly when their mobility was limited. One SU described his difficulty in navigating the health care system as such:

I had callouses on my feet from constant walking, itching, burning, and cracking. I tried not to go anywhere if I was sick because I did not want to spend money. I had a primary care physician, and then the primary care does not do foot care. He referred me to somewhere else [private doctor], and I could not get accepted without insurance. I struggle with how they treat me; I have experienced racism. So, I had to change doctors again and could not get my prescriptions. [HSU26]

HSUs also discussed their inability to promptly receive the appropriate care, which led to an unresolved and prolonged illness, loss of employment, and long-term disability. One HSU highlighted the long wait times when describing his experience of feeling vulnerable and seeking medical care for ongoing foot problems.

#### Theme 2: Lack of Trust and Fear of Discrimination

Lack of access to health care, mainly foot care services, often resulted in HSUs caring for their own feet in varying inside and outside environments. Extreme weather conditions, long periods of walking, and improper footwear contributed negatively to their foot health. One HSU summarized:

My feet are immersing in snow and are soggy. I catch my feet soaking in wet socks in the cold. I cannot take off my shoes; someone will steal them. I sleep in with my shoes on all day and night. I burnt my toes twice when I slept near to a campfire. I get cold feet and frostbite; sometimes, it is cold, wet, and moist. I have feet swelling, blisters, redness and drenched in the winter. I tried changing my socks ‘because I was in my shoes all day and I wanted to get out coz’ by that time it was soaked and trenched and had a nasty odour. I am embarrassed about my feet [shyly smiling]. I know it is hard for me to trust anyone because of my experiences. I had no insurance. So, I am not going to see the doctor until I am sick. I do not take the bus [mode of transportation] in some places. I cannot find a place to change my clothes or use a public washroom. I do not feel safe living outdoors at night. [HSU39]

Weather-related foot conditions prevented HSUs from seeking appropriate foot care as they expressed concerns about “*being embarrassed about feet”* [HSU12], having “*street feet”* [HSU27], a “*foul smell of feet”* [HSU18], or “*a frostbite”* [HSU24].

To keep their feet clean, dry, and odorless in the winter, some participants noted the importance of having dry places such as shelters that provide dry space for airing feet, access to clean water for bathing, foot care, and adequate waste disposal. While one HSU mentioned that he tries “*to look for dry places”* [HSU24], another mentioned that when he is outside, he must wear four to five socks, and therefore “*the shelter is important to [him] this winter to keep warm.*” [HSU1]. One study participant stated:

I have changed places, moved around, and have lived on my own. I live out of an abandoned vehicle, sleep here, just not my own home. I am constantly on the lookout for extra dry, warm clothes; they have food on some days at the centre [shelter]. I hold onto my [wet and dirty] socks and shoes in my baggage wherever I go. I do not like to go to the doctor [due to embarrassment], so I sometimes find this [Epsom salt] helps me. There is no real space to dry and warm my feet. [HSU56]

In addition to seeking out warm and dry places, others used self-led approaches to maintain foot health. One participant mentioned how she “*waited to find a place to remove [her] shoes and socks down by the river and wait for them to dry before [she] had shoes again. Sometimes [she] slept in [her] shoes.”* [HSU33]. Another woman stated that “*I sprinkle baby powder in my shoes and socks to help with the bad odour and foul smell of feet.”* [HSU18], while another stated that “*I get water in a little pot and just put water from the park and soak my feet in it. Without permanent shelters in the area, it is difficult. My options are limited.”* [HSU61].

Keeping the foot condition in good shape was important to participants. However, their inability to keep their feet clean, dry, and healthy slowed the healing and recovery of their foot problems, especially when their feet were exposed to long periods of walking. Homelessness affected the ability to walk, which was a primary mode of transport and work. They described more self-awareness regarding their foot care needs with this experience. They tend to walk “*every day”* [HSU15], “*all day from the time the time [they] wake up”* [HSU46], and “*walking an average of 3 to 4 miles a day”* [HSU53]. One HSU who stays in a shelter explained that during the day (9 am to 4 pm) when a person who is homeless must remain out of the shelter, he spends his time walking around the city:

My foot problems get worse. Being without shoes at night was not safe. I always had shoes on while I slept. I try to remove my shoes during the day to air out my feet. My feet easily tire out with walking for long distances on foot. I cannot walk on my left foot; my ankle hurts a lot. I limp on my left foot, which hurts my hip, lower back, and upper back. My feet are sore, especially on my left foot near the big toe and on the heel. I am exhausted when walking 13 miles each day. I feel lonely and insecure on the streets. [HSU36]

Another HSU discussed the impact of chronic homelessness, growing older, chronic pain, and loss of memory leading to missed appointments with health care providers, which in turn negatively affect his foot health.

HSUs also highlighted that improper footwear was a contributing factor to their existing and deteriorating foot conditions. One participant said, “*I have worn out footwear”* [HSU56]. Another HSU mentioned that he would “*prefer to trade his feet for a pair of new feet”* [HSU19]. However, another HSU explained that “*I never remove my shoes off; people [other HSUs] steal footwear. I have an old pair of footwear, and I constantly look out for a good pair of winter boots”* [HSU16]. When discussing their foot conditions considering footwear, one participant attributed her growing foot problems to improper footwear:

If I wear shoes all the time, the scars become more. I had sunburns on my feet from wearing sandals. I do not have the right footwear that fits my foot with my bunions and shark feet. I am going to the doctor for a better fit of shoes. My feet are bigger on the right side, 13 sizes, and 11 on the left. I had two different sizes of footwear. I look for dry shoes and socks each time. I cannot take off my shoes and socks. I keep my shoes and socks for 24 hours. If I keep them off, they [other SUs] will take them away. [HSU62]

One HSU mentioned that he would “*prefer to trade his feet for a pair of new feet”* [HSU19].

I am just getting my medication since I have had a road accident and injured my foot. I have been in and out of emergency. All I needed were the painkillers time to help with my pain. I do not get my usual painkillers; they tried another pain killer, which does not help with the pain. It knows about what my foot needs are and what is going to help it. I had bad nail fungus; I got apple cider vinegar to sit and soak my feet and fingernails. [SU51]

#### Theme 3: Need for Community Navigation and Social Support

Several HSUs expressed that HSUs face challenges with managing their foot conditions and maintaining foot health in daily lives, as one participant admitted: “*I do not know how to keep my feet dry and clean because I do not have a home. I understand that my feet take their unnatural course of events with time [seasons]. I wait for better days [warm summer days].”* [HSU39]. CSP expressed that engaging HSUs in their care and directing them to navigate health care resources helped these individuals. One CSP stated:

I have seen a few people who have had large open wounds due to blister formation from swelling, skin punctures, less circulation, weather-related conditions and swelling from diabetes. I am disappointed about how they speak about that person. While some people needed nail care, feet were not well cared for and had no proper support. One time a person visited the Shaman for foot healing, and then he came back. I guided them to find free wound clinics in the community for foot wounds and foot care. Some of them wait for a lift to get to another destination. I wish there were a simple way for them to access healthcare. [CSP3]

Another CSP discussed how they assist a person who is homeless in shelters by stating that:

We take care of them [HSUs]. Some of them [HSUs] clean their feet in the shower and air them out. Some of them [SUs] use foot lotions. If they need staff to remind them of their medication, we support residents. There are minimal services for follow up. There is a need to get involved with these populations that you work with to have compassion and empathy. We encourage them to go to the doctor, help connect them with a wound clinic or the ER if it is bad. Moreover, it is tough to be able to get a referral or maybe a specialist. They need more immediate access to care and support because they are vulnerable. [CSP1]

One CSP mentioned that although they have a few supplies for providing foot care, there is a lack of services that specifically focus on providing adequate foot care to HSUs. The CSP indicated they need to do more for this population:

We need to provide more dry and clean socks, clean shoes and boots, facilities for foot hygiene, foot powder, foot bandages, creams, nail care supplies, and access to nail care in the facility. I communicate about the essence [importance] of foot care; and provide encouragement, education, and referrals [to street nurses or the hospital emergency]. There is a need for help with wounds or referral, if needed, support, outreach for general health concerns and foot concerns. They [HSU] need the washing of the feet, treating wounds, airing feet, or directions on how to remedy foot issues. [CSP5]

CSP discussed how the social and health care initiatives are meeting the foot care needs of a person who is homeless. To explain these supports, one CSP mentioned that there were few health clinics in the community *“Some of the people [HSUs] are seen in the wound care clinic for their foot problems with referral”* [CSP4]. This CSP discussed the two health clinics at two different shelters, of which one is run by a “street nurse” while the other is not yet staffed by a health professional. The CSP also discussed a donor program by stating that *“We get extra winter socks, shoes, and coats for the people [HSUs] with community donors”* [CSP4].

While these community supports are available, one CSP indicated that “*Some people [HSUs] are not conscious of their feet, and dislike asking for help and feel unwelcome and shy. They refuse help and do things on their own, stay disconnected to the services, and stay out of the shelters”* [CSP6]. However, this was not the case for all *[HSUs]* with foot care needs. Another CSP mentioned that:

Some of the people [HSUs] are aware of the medical clinic, shelters, and know when to call 811 for non-emergency or 911 for emergency assistance. I think knowing the right services and who to ask for help and not be afraid to talk for seeking support is important. [CSP10]

CSP also recognized that more community support systems and resources are needed to meet *HSU*'s foot care needs. One CSP explained the challenges SUs face and the potential for a drop-in program that specifically caters to their foot care needs:

It is hard to get foot care or a foot examination when they [HSUs] have no address, no phone, no insurance, and no money to get foot treatment. They [HSUs] sometimes feel that their voice is not heard, and do not receive respect when they visit the medical clinic, and they feel rejected. There are few clinics in the community. Having a foot care clinic would help have foot care. [CSP9]

CSP's general message indicated that although some participants access the limited foot care services available to them, there is a need for more foot care programs to be implemented that have a greater reach in the community.

## Discussion

Despite the availability of health care services in small cities, HSUs expressed several barriers to accessing foot care services and remaining healthy. A lack of primary care results in increased utilization of emergency care services by HSUs. However, HSUs experienced discrimination because of their identity and status. HSUs described feelings of being invisible, ignored, disrespected, not getting the treatment they needed, and their requests not being acknowledged. They also described feeling stigmatized due to previous discriminatory experiences; and avoiding health care service usage due to embarrassment caused by poor personal hygiene. This finding is consistent with other studies that showed that HSUs face discrimination when accessing emergency services (Skosireva et al., 2014).

Discrimination contributes to long wait times and differential health access (Hwang et al., 2011), further increasing inequities and disparities (Stergiopoulos et al., 2016). For homeless individuals, mistrust of health care providers, stigma, and lack of access to primary care providers are barriers to accessing health care (Hwang et al., 2011). Social stigma causes an internalized self-blame, low self-esteem, and service users avoid using health care services (Campbell et. al., 2015; Hakanson & Ohlen, 2016). Crowded shelters, long periods of walking or standing and prolonged exposure of feet to moisture can lead to infections (Hwang et al., 2014). In our study, HSUs recognized that sleeping on the streets placed them at a risk for foot care problems and risked their comfort and safety as increasingly noted by female HSUs compared to male HSUs. The impact of not having a safe place to live, less access to resources, and traumatic history makes it difficult to access health services to address unmet foot care needs. Consequently, these barriers along with the lack of health insurance and difficulty navigating the health care system prevent HSUs from seeking help from the health care system.

Our study highlights that CSPs work with HSUs regardless of socioeconomic status, age, gender, and ethnic-racial identity. While HSUs generally have access to primary and urgent care, and street nurses for their general health care needs, their foot concerns continue to be overlooked as foot assessments are not part of the routine check-up (D'Souza et al., 2020). The situation is further worsened due to lack of access to proper and timely foot care, lack of health insurance, and lack of follow-up with a primary health care provider. Hence, foot care needs of HSUs go unmet.

According to a study by Frank et al. (2013), most HSUs do not have a follow-up appointment, which increases their likelihood of ending up in an emergency. Screening of study participants’ foot conditions showed that 72.31% had observed foot problems, while only 38.5% reported foot problems, requiring foot screening and objective measures of foot problems of service user’s (D’Souza et al., 2021). This lack of follow-up was also noted in our study; HSUs resorted to emergency services for their foot problems, and associated foot care needs were not followed up.

In our study, foot problems were more likely to result from improper footwear, increased moisture, and poor foot hygiene. Research on foot problems show that increased inadequate footwear and poor hygiene and self-management foot behaviors (D’Souza et al., 2015) lead to foot-related problems (Schwarzkopf et al., 2011). In their study, Muirhead et al. (2011) also observed that HSUs are more exposed to moisture, poor footwear, prolonged walking, poor foot hygiene, and repetitive trauma.

Our findings show that inattention to foot care was least prioritized because urgent priorities such as food, shelter, and clothing took precedence. Some of the participants did not have access to transportation (such as vehicles and public transit), health insurance, and government identification. However, they uncovered that it is challenging to navigate the health care system to obtain proper care for their health concerns. Our findings are consistent with other studies that have discussed the barriers that HSUs experience when accessing general health care services (Alecrim et al., 2016; Krausz & Schuetz, 2013), and the difficulty associated with navigating the health care system (Campbell et al., 2015).

The challenges of navigating the health care system were also reflected upon by the CSPs that it was vital for them to understand HSUs’ expectations of foot care to enable access and appropriate foot care tailored to each person's individual needs. CSPs perceived that better access to health care includes coordination of foot screening, timely scheduled appointments on the same day as needed to alleviate stress, maintaining frequent contact, and having a sensitive approach.

According to D’Souza et al. (2021), there is a link between low levels of education and chronic homelessness. Education, income, and nutritional status are determinants of health that, if addressed, may enhance foot care, and reduce foot complications (D’Souza et al., 2016). While several studies have discussed general health services for HSUs, foot care services are rarely discussed. This is reflected by existing studies that do not focus on foot care needs and, instead, discuss correlations between lower health literacy and poorer health outcomes (Berkman et al., 2011; Guyavarch & Le Méner, 2014).

Consistent with the SDHHE framework, we discovered that the complex interactions of several contributing individual factors to homelessness (lack of health care card, food insecurity, missed appointments, stigmatization, discrimination, and negative health care experiences) reduce HSUs’ access to equitable health care. We further discovered that system-based barriers (inadequate primary care providers, insufficient health insurance, lacking medical coverage, longer wait times, lack of transportation, and poor supportive housing) further contributed to this inequity. These individual and system-level barriers play a key role in contributing to poverty; and increasing the risk of becoming and remaining homeless. This exclusion, inequity, and lack of timely use of health care increases their unmet foot care needs.

It is important to help HSUs access appropriate health care in a timely manner, and for CSPs to appreciate how the social determinants of health impact HSUs’ access to equitable health services. When access to supportive housing, income, and jobs is a priority for HSUs, they are so focused on transition and survival that they tend to overlook their health care needs. This often increases their unmet health care needs that include foot screening, mobility devices, and appropriate medications. Therefore, CSPs should focus on helping HSUs in finding supportive and safe housing and receiving appropriate employment training. This is of particular importance for female HSUs who indicated safety concerns. According to the SDHHE framework, CSPs role in capacity building can help HSUs meet their basic needs and begin to focus on their general health care and foot care needs.

A holistic and a sensitive approach is needed to tackle health inequities that include actions that address the social structures and processes that systematically distribute the determinants of health unequally for service users. Community organizations and governance may be able to influence structural determinants and/or daily living conditions than service users. However, policy-level initiatives are needed to address key determinants of health, such as living environment, steady income, stable employment, better education, community support, healthy behaviors, and most importantly, supportive housing, and equitable access to health services. Only then will CSPs be better able to meet the care needs of HSUs. Since there is limited research on the challenges HSUs face in obtaining appropriate and timely care for foot problems, particularly in a small-city context, this study addresses this significant gap in the literature.

## Implications for Practice

The unmet foot care needs of HSUs are determined by their socioeconomic and housing status, which factor into their inability to access foot care services. Timely access to health care services can be provided to HSUs by extending daily clinic hours, improving transportation access, and designing specific foot care programs that take into consideration the various SDHHE that impact homeless individuals. There is a need to provide more accessible and diverse health care services by bringing health care services to the HSUs, such as by navigating with community providers and using mobile health clinics for outreach visits at multiple sites. While such mobile health clinics exist for cancer screening, drug testing, and needle exchanges, there are no such clinics for wound and foot care concerns. A mobile clinic focusing on assessments, diagnosis, and interventions for foot-related concerns can help to reduce the severity of vascular foot problems, amputation, and other long-term foot-related complications. In addition to mobile clinics, improvising health clinics in drop-in programs and shelters will improve access to health care, enable health care providers to build trust, and enable CSPs to build rapport with HSUs.

We recommend the need for holistic strategies and navigation services to reach out to HSUs and increase community and health care resources to facilitate foot care needs. Timely and appropriate access to mobile clinics can promote foot health, prevent complications and illnesses, and improve HSUs’ access to health services. Such clinicals will enable health professionals (e.g., outreach nurses) to screen, detect, and assess foot, vascular, and skin problems among HSUs so that interventions can be implemented promptly to reduce long-term complications, emergency visits, and hospitalizations. By collaborating with community and health services, outreach programs can play an essential role in improving HSUs’ quality of life. Such programs could provide HSUs with foot accessories such as clean and dry socks, shoes, and boots; clean hygiene materials such as clean clothing, bowls with warm water, good nail care and sanitization kits, moisturizers, and minor dressings and bandages. This collaboration could further ensure that HSUs receive timely referrals to appropriate health care providers.

We recommend that community agencies collaborate with primary health care and optimize resources to meet the health care needs of HSU. There is a need to clarify the role of the health care system in supporting multidisciplinary, collaborative care for HSU's health care needs, particularly for those who currently live with foot conditions and those who are at risk for developing foot problems. Setting up mobile clinics, extending clinic hours on weekends, involving community nurses to deliver primary foot care guidance and treatment, and building social support networks associated with homelessness can facilitate equity and better access to health care specialists and podiatry services. This strategy would ensure that foot care issues are resolved, hospital and emergency visits reduced, and the quality of life enhanced for HSUs whose health care needs are often overlooked. Ideally, such foot care strategies should prevent or delay foot problems, provide opportunities for health care professionals to recognize people at increased risk of developing foot disease, and ensure that HSUs with complex illnesses receive appropriate care that is suitable for their individual foot care needs.

Based on the SDHHE, it is essential to ensure service users have equitable access to public services that contribute to health equity, and that the spending on services is equitable. Our study indicates that several social determinants of health and health equity impact HSUs. These include low education, poor physical environments, poor social support, unhealthy behaviors, lack of access to health services, and gender. Their views are often not considered during health care planning, nor are they involved in decision making about health care services. Our study aimed to engage with this population to understand their viewpoints on the state of foot care services to create opportunities to influence innovation and restructuring of health services that could meet HSU's foot care needs. To bring HSU's perceptions to the forefront and ensure equitable access to health care services, CSPs need to work closely with homeless individuals and include them in health care decisions and involve them when building meaningful community health partnerships.

## Limitation

Our findings support the validation and triangulation of multiple stakeholders’ voices through a community-oriented approach that illustrates the need for enhanced coordination of equitable health care services for HSUs. However, there were some limitations to this study. The study did not address that the number of people who are homeless are indigenous, and the systemic issues that impact their health and health care. We utilized a small sample size of HSUs and CSPs. We used one-on-one interviews and could have considered focus group discussions to allow for more in-depth discussions and multiple perspectives on the prominent barriers commonly experienced by HSUs in general. Furthermore, we did not focus on the psychosocial, emotional, or mental health experiences of HSUs.

## Conclusion

People experiencing homelessness are vulnerable and express unmet foot care concerns that are often overlooked by the health care system. Ensuring HSUs have equitable access to health care and regular contact with community health services is essential when addressing foot care needs. Our study provides insight into HSUs’ experiences with accessing health care services for their unmet foot care needs; and the experiences of CSPs who try to coordinate health and community services for this population. The study findings show that HSUs with foot problems receive inequitable care and face challenges in accessing appropriate and timely care, which contributes to increased emergency visits, loss of trust in the health care system, and deterioration in health. CSPs in the community and within the health care system must be responsive and compassionate to HSUs’ foot care needs. By playing a proactive role, CSPs can identify specific foot problems, collaborate with other sectors to strategize, and prioritize innovative solutions to improve HSUs’ foot health. While foot care services are readily available in larger urban centers, there is a dire need for foot care services in small cities surrounded by rural and remote areas.
